# Activated Graphene Deposited on Porous Cu Mesh for Supercapacitors

**DOI:** 10.3390/nano11040893

**Published:** 2021-03-31

**Authors:** TaeGyeong Lim, TaeYoung Kim, Ji Won Suk

**Affiliations:** 1 School of Mechanical Engineering, Sungkyunkwan University, Suwon 16419, Gyeonggi-do, Korea; taegyung95@gmail.com; 2 Department of Materials Science and Engineering, Gachon University, Seongnam 13120, Gyeonggi-do, Korea; taeykim@gachon.ac.kr; 3 Department of Smart Fab. Technology, Sungkyunkwan University, Suwon 16419, Gyeonggi-do, Korea; 4 SKKU Advanced Institute of Nanotechnology (SAINT), Sungkyunkwan University, Suwon 16419, Gyeonggi-do, Korea

**Keywords:** copper mesh, ammonia, activated graphene, supercapacitor

## Abstract

A porous Cu (P-Cu) mesh was used as a current collector and its morphological effect on the supercapacitor performance was investigated. A porous surface was obtained by thermally annealing the Cu mesh using ammonia gas. Hierarchically porous activated graphene (AG) with a high specific surface area (SSA) was deposited on the P-Cu mesh using electrophoretic deposition, aided by graphene oxide (GO). GO was thermally converted to electrically conductive reduced graphene oxide (rGO). The AG/rGO that was deposited on the P-Cu mesh achieved a high specific capacitance of up to 140.0 F/g and a high energy density of up to 3.11 Wh/kg at a current density of 2 A/g in 6 m KOH aqueous electrolyte. The high SSA of AG and the porous surface morphology of the Cu mesh allowed efficient electric double-layer formation and charge transport. This work offers an alternative to improve supercapacitors by combining a porous metallic current collector with porous AG.

## 1. Introduction

Supercapacitors have attracted great attention in recent years as energy storage devices owing to their high power density, fast charge and discharge rates, long cycle life, and relatively simple structures [1,2]. However, their wide applications have been limited because their energy density is lower than that of conventional secondary batteries [3,4]. To enhance the performance of supercapacitors, most studies have focused on the development of high-performance electrode materials [5,6]. Graphene-based materials have been widely used as electrode materials due to their high specific surface area (SSA), high electrical conductivity, and thermal stability at moderate temperatures [7,8,9]. In particular, activated graphene (AG), which is synthesized by KOH-based chemical activation of graphene, has recently been reported to reach an extremely high SSA value of up to ~3100 m^2^/g with hierarchically porous structures [10,11]. Therefore, AG has been extensively investigated as an electrode material for advanced supercapacitors such as flexible and fiber-shaped supercapacitors with high performances [12,13].

In addition to the electrode materials, several studies have been conducted to improve the current collectors in supercapacitors. For example, the interfacial contact between the active electrode material and current collector was improved by incorporating carbonaceous materials [14], graphite inks [15], nanowires [16], and vertical graphene [17] on the current collector. Furthermore, various forms of current collectors, such as metallic wires and mesh, have been developed for energy storage devices with different shapes [13,18]. For instance, metallic-mesh electrodes have been used to develop planar supercapacitors, which demonstrated flexibility and transparency [19,20].

In this work, to improve the performance of supercapacitors using metal mesh, we utilized a porous Cu (P-Cu) mesh as a current collector and highly porous AG as an active electrode material. The surface of a commercially available Cu mesh was etched by thermal annealing using ammonia (NH_3_) gas. Hierarchically porous AG and graphene oxide (GO) were deposited on the P-Cu mesh surface using electrophoretic deposition (EPD). Subsequent thermal annealing was performed to convert the GO into electrically conductive reduced graphene oxide (rGO). The AG/rGO, which was deposited on the P-Cu mesh, was electrochemically tested for supercapacitors.

## 2. Materials and Methods

### 2.1. Development of P-Cu Mesh Using Ammonia-Gas Etching

The P-Cu mesh was obtained by thermally annealing a Cu mesh (wire diameter = 160 μm, 50 mesh, Nilaco Corporation, Tokyo, Japan) using ammonia gas. The raw Cu mesh was cleaned using 0.1 m ammonium persulfate for 1 min and rinsed with deionized water. It was annealed using hydrogen (50 sccm) and argon (200 sccm) at 1000 °C for 30 min. Then, ammonia (20 sccm) without hydrogen was introduced for 20 min to etch the Cu mesh surface.

### 2.2. AG Synthesis

To synthesize AG, chemical activation by KOH was performed using rGO powders (rGO-V50, Standard Graphene, Ulsan, Korea) [10,13]. The KOH and rGO powders were mixed in water by stirring (rGO/KOH weight ratio = 1/8). The mixture was placed in an alumina boat and dried in an oven at 120 °C for 24 h. Subsequently, it was loaded into a tube furnace and annealed at 800 °C for 1 h under an argon atmosphere [10]. AG was washed using acetic acid (10% water) and dried in an oven at 100 °C for 24 h.

### 2.3. Deposition of AG/rGO on the Cu Mesh

AG was deposited on the Cu mesh using EPD [13]. GO (GO-P, Grapheneall, Siheung, Korea) and AG were mixed in deionized water at a weight ratio of 1:1. EPD was performed on the AG/GO aqueous suspension using a two-electrode system where a Cu mesh and a Pt plate were used as working and counter electrodes, respectively. A 10 V voltage was applied on the working electrode for 1 min with mild stirring. The Cu mesh, which was coated with AG/GO, was dried for 24 h. The sample was thermally annealed at 600 °C for 1 h to obtain AG/rGO, which was deposited on the Cu mesh.

### 2.4. Electrochemical Testing

To test the electrochemical performance of the synthesized AG material, AG was mixed with 5 wt.% polytetrafluoroethylene (60 wt.% dispersion in water, Sigma Aldrich, St. Louis, MO, USA) binder to assemble the electrode [11]. The mixture was homogenized and rolled to form a 50 μm thick sheet. The electrodes were prepared by punching the sheet into circular disks with 1 cm diameter. The supercapacitor test cells were assembled in a symmetric two-electrode configuration with two current collectors (conductive films, z-flo 2267P, Transcontinental Advanced Coatings, Matthews, NC, USA), two electrodes, a porous separator (3501, Celgard, Charlotte, NC, USA), and a 6 m KOH aqueous electrolyte. The test cell was supported by two stainless steel plates [11].

The AG/rGO-coated Cu meshes were cut into 1 cm × 1 cm size and had a mass of 0.7 mg. The sample was used as a current collector and an electrode material without any binder. The AG/rGO-coated Cu mesh was electrochemically tested using the same apparatus without any additional current collector.

Electrochemical tests of the assembled symmetric supercapacitors were performed by cyclic voltammetry (CV), galvanostatic charge/discharge (GCD), and electrochemical impedance spectroscopy (EIS) using a potentiostat (Autolab PGSTAT204, Metrohm, Herisau, Switzerland).

The specific capacitance ($C_{m}$) of a single electrode was calculated from the GCD curves using the following equation [13,21]:

$$C_{m}=\frac{2I}{m\;dV/dt},$$

where I (A) is the discharge current, dV/dt (V/s) is calculated from the slope of the linearly fitted discharge curve, and m is the mass of the single electrode. The current density in GCD tests was obtained by dividing the applied current by the mass of two electrodes.

The specific capacitance ($C_{m}$) of a single electrode was calculated from the CV curves using the following equation [13]:

$$C_{m}=\frac{2\int I\left(V\right)dV}{mv∆V},$$

where I (A) is the current, V (V) is the applied voltage, v (V/s) is the scan rate, ∆V (V) is the total scanning voltage, and m is the mass of the single electrode.

The gravimetric energy density ($E_{m}$) and powder density ($P_{m}$) of the device were evaluated from the GCD curve using the following equation [13,21]:

$$E_{m}=\;\frac{1}{8}C_{m}U^{2},$$

$$P_{m}=\;\frac{E_{m}}{∆t_{discharge}},$$

where U (V) is the discharge potential and $∆t_{discharge}$ (s) is the discharge time.

### 2.5. Characterization of the Materials

The morphology of the Cu mesh and AG/rGO was observed using scanning electron microscopy (SEM, JSM7000F, JEOL, Tokyo, Japan). The chemical composition and structure of AG/rGO were characterized by X-ray photoelectron spectroscopy (XPS, ESCALAB250, Thermo Fisher Scientific, Waltham, MA, USA) using monochromated Al K_α_ radiation and Raman spectroscopy (XperRam35V, Nanobase, Seoul, Korea) with a 405-nm excitation laser. In the XPS analysis, peak deconvolution of the C 1s core-level spectrum was performed using the asymmetric Doniach–Sunjic line shape for the sp^2^-hybridized carbon, Gaussian–Lorentzian functions for the other spectral components, and the Shirley background model [22,23,24]. The nitrogen adsorption–desorption isotherms of AG were measured at 77 K (BELSORP-mini II, MicrotracBEL, Osaka, Japan).

## 3. Results and Discussion

### 3.1. Fabrication and Characterization of the AG/rGO-Coated P-Cu Mesh

**Figure 1:**
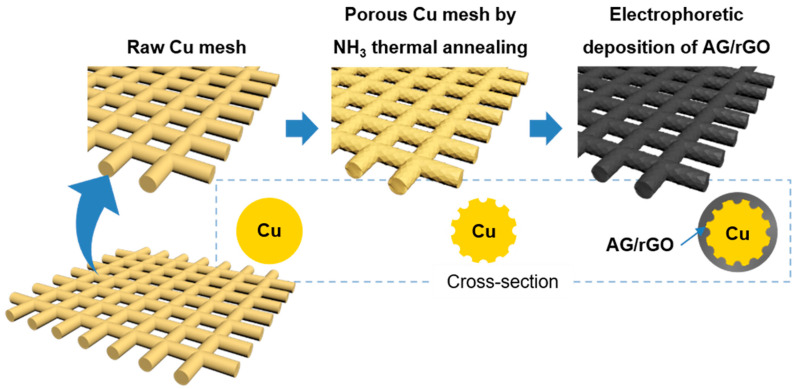
Schematic illustration of the fabrication of activated graphene (AG)/reduced graphene oxide (rGO) deposited on a porous Cu (P-Cu) mesh.

**Figure 2:**
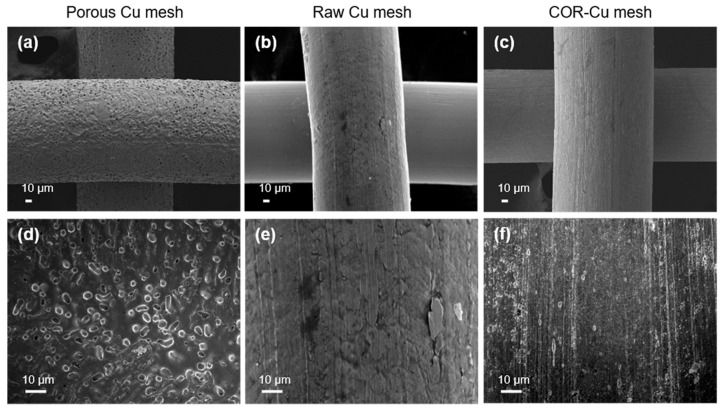
SEM images of (**a**,**d**) P-Cu, (**b**,**e**) raw Cu (R-Cu), and (**c**,**f**) bare Cu (B-Cu) meshes. (**d**–**f**) High-magnification images of each Cu mesh.

The fabrication process of the P-Cu mesh coated with AG/rGO is schematically shown in Figure 1. Ammonia was used as a copper etchant for the integrated-circuit fabrication and synthesis of the nanoclusters [25,26]. In this work, the Cu-mesh surface morphology was modified by the gas-phase ammonia treatment. The P-Cu mesh was obtained by annealing the Cu mesh in ammonia atmosphere at 1000 °C. The ammonia-etching process generated rough and porous surfaces on the P-Cu mesh (Figure 2a,d), as compared with the smooth surface of the raw Cu (R-Cu) mesh (Figure 2b,e). In addition, the copper oxide formed on the Cu mesh was chemically removed by immersing the Cu mesh in 0.1 m ammonium persulfate for 1 min. The bare Cu (B-Cu) mesh without a native oxide exhibited slightly rough surfaces due to the chemical etching by ammonium persulfate (Figure 2c,f).

**Figure 3:**
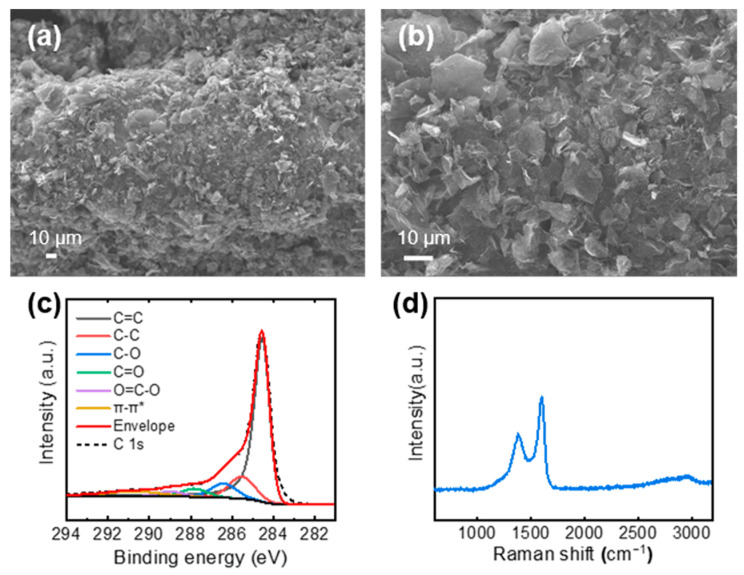
(**a**,**b**) SEM images of the AG/rGO-coated P-Cu mesh at (**a**) low and (**b**) high magnification. (**c**) XPS C 1s spectra and (**d**) Raman spectrum of AG/rGO.

AG, which was synthesized by the KOH-based chemical activation, was deposited on the Cu mesh using EPD [13]. To promote EPD of the AG, it was mixed with GO. AG/GO, which was deposited on the Cu mesh, was thermally annealed at 600 °C to convert the insulating GO into electrically conductive rGO. Therefore, rGO worked as a binder of the AG as well as an active electrode material for the supercapacitors. The surface of the AG/rGO/P-Cu mesh showed uniformly distributed AG and rGO on the P-Cu mesh (Figure 3a,b). XPS and Raman spectroscopy were used to investigate the chemical composition of the AG/rGO active material. The C 1s spectrum of the XPS was deconvoluted into spectral components of the sp^2^-hybridized carbon (C=C), sp^3^-hybridized carbon (C–C), C–O, C=O, O=C–O, and π–π* transition located at binding energy values of 284.6, 285.5, 286.4, 287.8, 289.0, and 290.7 eV, respectively (Figure 3c) [24,27,28]. The XPS analysis confirmed that AG/rGO demonstrated a high carbon content with a C/O ratio of 4.33. The Raman spectrum of AG/rGO exhibited typical characteristics of rGO with the presence of the strong D and G bands positioned at approximately 1350 and 1580 cm^−1^, respectively (Figure 3d) [29].

### 3.2. Pore Structures and Electrochemical Testing of AG

**Figure 4:**
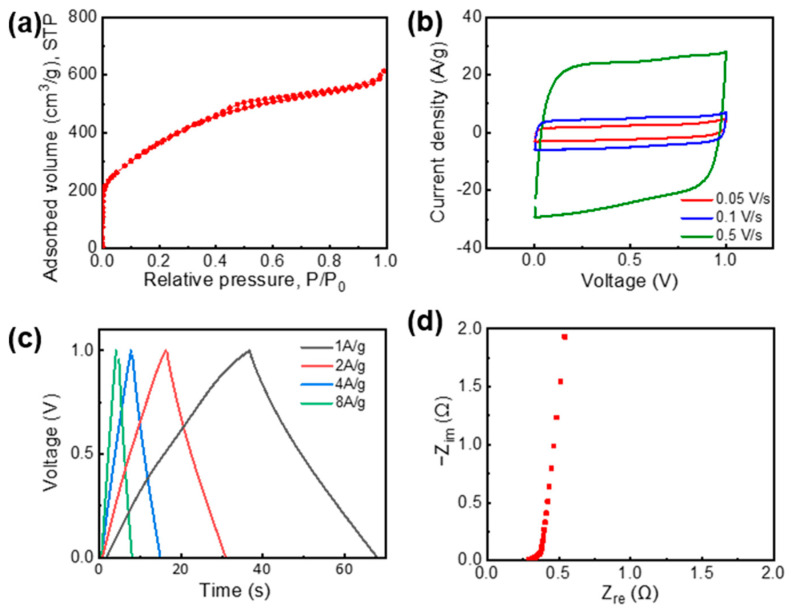
Pore characteristics and electrochemical performance of AG. (**a**) Nitrogen adsorption–desorption isotherms. (**b**) Cyclic voltammetry (CV) curves at scan rates of 0.05, 0.1, and 0.5 V/s. (**c**) Galvanostatic charge/discharge (GCD) curves at current densities from 1 to 8 A/g. (**d**) Nyquist plot.

Because the pore structure of electrode materials greatly affects the electrochemical performance of supercapacitors, the nitrogen adsorption–desorption isotherms were measured to characterize the AG porosity. Figure 4a shows that AG exhibited the characteristics of type IV isotherms in the International Union of Pure and Applied Chemistry (IUPAC) classification, which indicated the presence of a large fraction of mesopores [30,31]. The SSA value of AG, which was calculated using the Brunauer–Emmett–Teller method, reached up to 1339 m^2^/g. Thus, AG exhibited hierarchically porous structures with large amounts of micro- and mesopores, which facilitated the fast electrolyte ion transport as well as demonstrated a high charge-storage capability [10].

The electrochemical performance of the synthesized AG was measured using a 6 m KOH aqueous electrolyte with a symmetric two-electrode supercapacitor configuration. The CV curves showed a rectangular shape at scan rates from 0.05 to 0.5 V/s (Figure 4b). The GCD curves exhibited triangular shapes with good symmetry at current densities of 1, 2, 4, and 8 A/g (Figure 4c). These results indicate a good electric double-layer (EDL) formation of AG. Therefore, the AG electrode demonstrated high specific capacitances of 133 and 121 F/g at current densities of 1 and 2 A/g, respectively. In addition, the Nyquist plot in the frequency range from 50 kHz to 0.1 Hz featured a vertical line in the low-frequency region, which indicated an almost ideal capacitive behavior of AG. In this regard, synthesized AG demonstrated high electrochemical performance for supercapacitors.

### 3.3. Electrochemical Performance of the AG/rGO-Coated Cu Mesh

**Figure 5:**
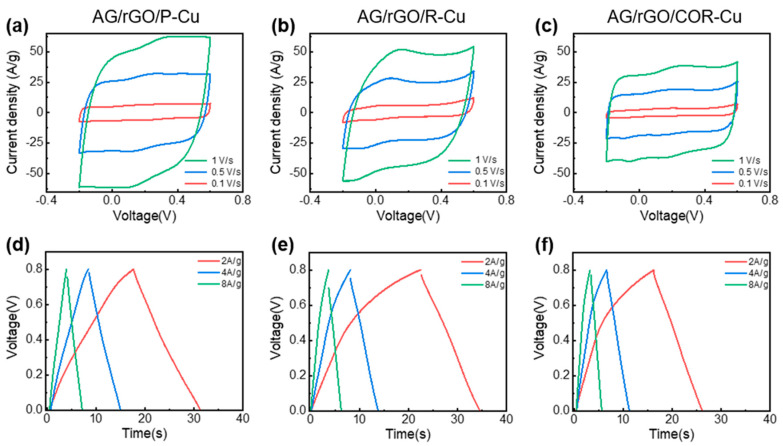
(**a**–**c**) CV curves of the supercapacitors using AG/rGO deposited on (**a**) P-Cu, (**b**) R-Cu, and (**c**) B-Cu mesh electrodes at scan rates of 0.1, 0.5, and 1 V/s. (**d**–**f**) GCD curves of the supercapacitors using AG/rGO deposited on (**d**) P-Cu, (**e**) R-Cu, and (**f**) B-Cu mesh electrodes at current densities of 2, 4, and 8 A/g.

The symmetric supercapacitors were constructed using two identical AG/rGO-coated Cu meshes and a 6 m KOH aqueous electrolyte. The effect of the P-Cu mesh on the electrochemical performance of the supercapacitor was compared with those of the supercapacitors that used two different Cu meshes: R- and B-Cu meshes. AG/rGO was deposited on these three Cu meshes for electrochemical testing. Figure 5a–c shows the CV curves of the supercapacitors that used the AG/rGO-coated Cu meshes at scan rates of 0.1, 0.5, and 1 V/s. The shapes of the CV curves were almost rectangular because of the outstanding EDL behavior of AG. However, the CV profile of the AG/rGO/R-Cu mesh displayed a relatively distorted shape, which could be attributed to the redox reaction on the Cu native oxide layer [32,33]. The specific capacitances of the P-, R-, and B-Cu meshes coated with AG/rGO at a scan rate of 0.1 V/s were 114.3, 98.6, and 59.4 F/g, respectively.

Figure 5d–f shows the GCD curves of the supercapacitor that used the AG/rGO-coated Cu meshes at current densities of 2, 4, and 8 A/g. The GCD curve of the AG/rGO/P-Cu mesh exhibited symmetric and linear shapes, whereas the R- and B-Cu meshes coated with AG/rGO showed relatively asymmetric and non-linear GCD curves. The specific capacitances of the P-, R-, and B-Cu meshes coated with AG/rGO at a current density of 2 A/g were 140.0, 122.1, and 100.6 F/g, respectively. The AG/rGO/P-Cu mesh showed an improved specific capacitance, compared with that of AG tested at the same current density of 2 A/g. This specific capacitance value of the AG/rGO/P-Cu mesh is higher than or comparable to those reported in other works that used metallic meshes as current collectors; for example, the previous works have shown specific capacitances of 63 F/g for oxidized single-walled carbon nanohorn/nanotube composites on Pt mesh [34], 107.8 F/g for carbon ink coated on Ni/Au-deposited stainless steel mesh [35], 152 F/g for mesoporous carbon nanofibers on Ni mesh [36], and 156 F/g for biomass-derived activated carbon on stainless steel mesh [37].

Moreover, the equivalent series resistances of the P-, R-, and B-Cu meshes estimated from the IR drop in the discharge curve at a current density of 2 A/g were 1.88, 4.03, and 1.11 Ω, respectively. This result implies that the removal of the Cu oxide layer in the P- and B-Cu meshes reduced the interfacial resistances of the device. Because of the outstanding electrochemical performance of the AG/rGO/P-Cu mesh, the device composed of the AG/rGO/P-Cu mesh exhibited the highest energy density of 3.11 Wh/kg and power density of 0.83 kW/kg. In contrast, the R- and B-Cu meshes demonstrated relatively lower energy densities of 2.71 and 2.46 Wh/kg, respectively.

**Figure 6:**
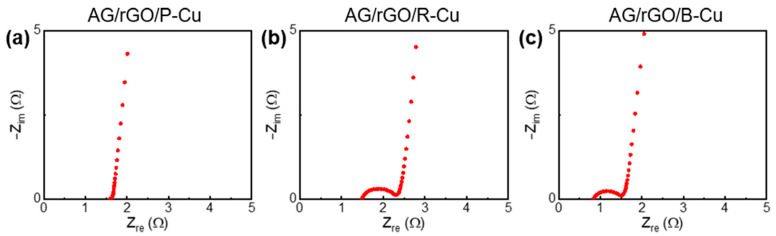
Nyquist plots of the supercapacitors using AG/rGO deposited on (**a**) P-Cu, (**b**) R-Cu, and (**c**) B-Cu mesh electrodes. The frequency ranges from 50 kHz to 0.1 Hz.

EIS analysis was performed to further investigate the effect of the P-Cu mesh. The Nyquist plots of the AG/rGO-coated Cu meshes were obtained in the frequency range from 50 kHz to 0.1 Hz (Figure 6). The semicircle diameter in the high-frequency region of the Nyquist plots corresponded to the charge-transfer resistance ($R_{ct}$) related to the electrode resistance, contact between the electrode and current collector, and electrolyte ionic resistance inside the pores of the electrode [38,39]. $R_{ct}$ of the AG/rGO/P-Cu mesh was almost 0 Ω, whereas those of the R- and B-Cu meshes with AG/rGO were 0.81 and 0.67 Ω, respectively. This result indicates that the P-Cu mesh with a porous surface provided improved contacts between the electrode materials and current collector surface, which led to efficient charge transfer with rapid and excellent EDL formation on the AG/rGO surfaces [38,39,40,41].

**Figure 7:**
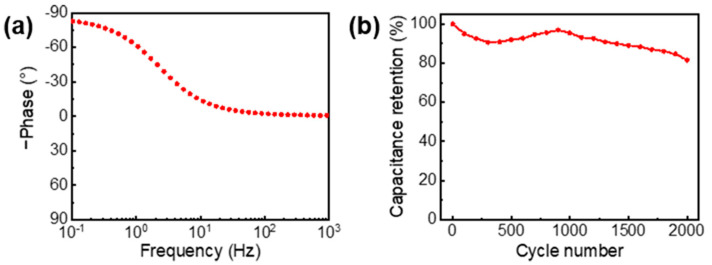
(**a**) Impedance phase angle versus frequency plot of the AG/rGO/P-Cu mesh electrode. (**b**) Cycling stability test of the AG/rGO/P-Cu mesh supercapacitor at a current density of 4 A/g for 2000 cycles.

Moreover, the capacitive characteristics of the AG/rGO/P-Cu mesh was confirmed by the impedance phase angle plot as shown in Figure 7a. The phase angle was nearly −90° at low frequencies, which is indicative of capacitive behaviors [11]. The supercapacitor using the AG/rGO/P-Cu mesh also exhibited the stable and long cycling performance with approximately 82% capacitance retention at a current density of 4 A/g after 2000 cycles (Figure 7b).

## 4. Conclusions

A P-Cu mesh, which was obtained by etching a Cu mesh using ammonia gas at a high temperature, was used as a current collector in supercapacitors. Highly porous AG, which was synthesized by KOH-based chemical activation, was deposited on the P-Cu mesh using EPD. The combination of the P-Cu mesh and AG provided a high capacitance of up to 140.0 F/g, which corresponded to an energy density of 3.11 Wh/kg and power density of 0.83 kW/kg. This is due to the excellent charge transport and EDL formation of the AG/rGO/P-Cu mesh. This study provides an alternative method for enhancing the current collector and electrode materials for advanced supercapacitors.
